# Preoperative radiotherapy in patients with locally advanced esophageal squamous cell carcinoma: a narrative review

**DOI:** 10.3389/fonc.2025.1613954

**Published:** 2025-07-22

**Authors:** Yan Lin, Shou-Feng Wang, Huan-Wei Liang, Yang Liu, Wei Huang, Xin-Bin Pan

**Affiliations:** ^1^ Department of Gastroenterology, Jiangbin Hospital of Guangxi Zhuang Autonomous Region, Nanning, Guangxi, China; ^2^ Department of Thoracic Surgery, Guangxi Medical University Cancer Hospital, Nanning, Guangxi, China; ^3^ Department of Radiation Oncology, Guangxi Medical University Cancer Hospital, Nanning, Guangxi, China

**Keywords:** esophageal cancer, surgery, radiotherapy, chemotherapy, immunotherapy

## Abstract

Neoadjuvant treatments play a crucial role in improving survival rates for patients with locally advanced resectable esophageal cancer. The CROSS and NEOCRTEC5010 trials have shown that neoadjuvant concurrent chemoradiotherapy significantly enhances survival compared to surgery alone. In contrast, the NeoRes and CMISG1701 trials indicate that while neoadjuvant chemoradiotherapy yields a higher histological complete response rate, it does not confer survival benefits over neoadjuvant chemotherapy. The recent JCOG1109 trial has demonstrated that neoadjuvant triplet chemotherapy offers a statistically significant overall survival advantage compared to doublet chemotherapy. However, combining doublet chemotherapy with radiotherapy did not show notable survival improvement compared to doublet chemotherapy alone. Additionally, neoadjuvant immunotherapy in conjunction with chemotherapy has shown a greater histological complete response rate compared to neoadjuvant chemotherapy, with comparable rates to neoadjuvant chemoradiotherapy. These findings have sparked debate regarding the necessity of radiotherapy in neoadjuvant treatment protocols. This review aims to elucidate the role of radiotherapy based on the current evidence and to assess ongoing and future trials that may address existing knowledge gaps. It will also underscore the challenges in making definitive recommendations about radiotherapy, particularly as technologies and treatment modalities continue to advance.

## Introduction

Esophageal cancer is the sixth most commonly diagnosed cancer and the fifth leading cause of cancer-related deaths globally, representing a significant public health burden (1). Esophageal squamous cell carcinoma predominates in East Asia, while adenocarcinoma is more common in Western countries. Unfortunately, nonspecific symptoms often lead to diagnosis at an advanced stage (2). Consequently, many patients with locally advanced disease are initially deemed unresectable. However, a subset with localized disease can achieve cure through neoadjuvant therapy followed by surgery.

## Neoadjuvant chemotherapy as an alternative to chemoradiotherapy

Neoadjuvant chemoradiotherapy followed by surgery is the established standard for locally advanced resectable esophageal cancer, regardless of histological subtype (3). The CROSS trial demonstrated that neoadjuvant chemoradiotherapy significantly improves overall survival compared to surgery alone in patients with locally advanced adenocarcinoma or squamous cell carcinoma (hazard ratio [HR] = 0.70, 95% confidence interval [CI]: 0.55-0.89; P = 0.004) (4, 5). Similarly, the NEOCRTEC5010 trial confirmed the survival benefit of neoadjuvant chemoradiotherapy over surgery alone in locally advanced esophageal squamous cell carcinoma (HR = 0.74, 95% CI: 0.57-0.97; P = 0.03) (6, 7).

Despite this evidence, clinical adoption of neoadjuvant chemoradiotherapy remains limited, with fewer than 5% of patients in China receiving this treatment (8). Several factors contribute to this low uptake. Firstly, while over 50% of esophageal cancer patients in high-income countries receive radiotherapy for local control, access is significantly constrained in low- and middle-income countries (9). Radiotherapy infrastructure is often underdeveloped in these regions, limiting global accessibility for esophageal cancer patients (10).

Secondly, multidisciplinary team discussions are crucial for managing esophageal cancer, particularly in formulating radiotherapy-based strategies. However, only a small proportion of patients are reviewed in multidisciplinary team meetings, which are vital for optimizing staging, ensuring timely surgery, and improving adherence to clinical guidelines (11–13).

Thirdly, some surgeons express concern that neoadjuvant chemoradiotherapy may increase toxicity, potentially leading to greater operative difficulty, higher postoperative morbidity, and mortality (14–18). Additionally, disease progression during neoadjuvant chemoradiotherapy or patient preference may prevent some patients from proceeding to surgery (19).

Given these challenges, neoadjuvant chemotherapy is increasingly recognized as a viable alternative to chemoradiotherapy, offering comparable survival outcomes. The NeoRes trial found no significant difference in 3-year overall survival between the two approaches for locally advanced esophageal or junctional cancer (HR = 1.11, 95% CI: 0.74-1.67; P = 0.77) (20). Similarly, the CMISG1701 trial revealed no significant survival difference between the two therapies in esophageal squamous cell carcinoma patients (HR = 0.82, 95% CI: 0.58-1.18; P = 0.28) (21). Based on this evidence, neoadjuvant radiotherapy may not be essential (22).

Despite the use of neoadjuvant chemotherapy followed by esophagectomy, the prognosis for locally advanced resectable esophageal cancer remains poor, with 5-year survival rates around 50-60%. Furthermore, many patients undergoing radical esophagectomy experience postoperative complications, hindering the completion of adjuvant chemotherapy (23). Consequently, more intensive neoadjuvant strategies are needed to improve long-term outcomes.

Summary: Neoadjuvant chemotherapy may be an alternative to standard chemoradiotherapy, particularly where radiotherapy access or multidisciplinary coordination is limited, as trials show comparable survival. However, despite this alternative approach, overall survival remains poor and postoperative complications hinder further treatment, necessitating more intensive neoadjuvant regimens.

## Intensive neoadjuvant chemotherapy enhances survival outcomes

The JCOG1109 trial evaluated the efficacy and safety of triplet chemotherapy (fluorouracil, cisplatin, and docetaxel) compared to doublet chemotherapy (fluorouracil and cisplatin) and chemoradiotherapy (41.4 Gy combined with fluorouracil and cisplatin) in patients with locally advanced esophageal squamous cell carcinoma (24). Results showed that triplet chemotherapy significantly improved the 3-year overall survival rate compared to doublet chemotherapy (HR = 0.68, 95% CI: 0.50-0.92; P = 0.006). However, no significant difference was observed between chemoradiotherapy and doublet chemotherapy (HR = 0.84, 95% CI: 0.63-1.12; P = 0.12). Although triplet chemotherapy showed a numerically better survival trend compared to chemoradiotherapy, this difference was not statistically significant (HR = 0.80, 95% CI: 0.59-1.10; P > 0.05).

Nevertheless, intensive neoadjuvant chemotherapy is associated with increased adverse events. Grade ≥3 febrile neutropenia occurred in 1%, 16%, and 5% of patients in the doublet chemotherapy, triplet chemotherapy, and chemoradiotherapy groups, respectively. Grade ≥2 postoperative pneumonia, anastomotic leak, and recurrent laryngeal nerve paralysis were reported in the doublet group (10%, 10%, 15%), triplet group (10%, 9%, 10%), and chemoradiotherapy group (13%, 13%, 10%).

Similarly, the ESOPEC trial, a two-arm randomized phase III study, compared perioperative chemotherapy (5-FU, leucovorin, oxaliplatin, and docetaxel) with neoadjuvant chemoradiation (41.4 Gy plus carboplatin and paclitaxel) (25, 26). After a median follow-up of 55 months, the 3-year overall survival rate was significantly higher in the chemotherapy group (57.4% vs. 50.7%). This survival advantage persisted at 5 years (50.6% vs. 38.7%). The chemotherapy regimen reduced the risk of death by 30% (HR = 0.70, 95% CI: 0.53-0.92; P = 0.012).

Collectively, these trials suggest that intensive neoadjuvant chemotherapy could become the new standard of care for locally advanced esophageal cancer, potentially obviating the need for radiotherapy in neoadjuvant strategies.

Summary: The JCOG1109 and ESOPEC trials indicates that intensive neoadjuvant chemotherapy regimens significantly enhance survival outcomes compared to standard doublet chemotherapy or chemoradiotherapy, despite increased toxicity risks, suggesting its potential as a new standard that may obviate the need for radiotherapy.

## Reasons for survival improvement with intensive neoadjuvant chemotherapy

Intensive neoadjuvant chemotherapy facilitates tumor downstaging, improves R0 resection rates, and helps eliminate potential micrometastases and occult distant lesions. The CROSS trial reported comparable distant metastasis rates between surgery alone and neoadjuvant chemoradiotherapy (27% vs. 28%) over 10 years of follow-up (5). In contrast, the JCOG1109 trial demonstrated a lower incidence of distant-only recurrences with triplet chemotherapy versus chemoradiotherapy (31.6% vs. 49.3%) (24).

Notably, locoregional-only recurrences occurred more frequently in the triplet chemotherapy group (43.4% vs. 22.7%). Patients with locoregional recurrence in this cohort were more likely to receive subsequent chemoradiotherapy (50.0% vs. 17.1%) or radiotherapy alone (28.4% vs. 21.4%), indicating better access to curative-intent salvage therapies. Consequently, locoregional-only recurrence patients in the triplet group experienced longer survival (18.9 vs. 9.9 months).

These findings suggest that while locoregional recurrence may be manageable with curative radiotherapy, long-term survival depends more critically on controlling distant metastases, a goal potentially better achieved through intensive systemic therapy. In JCOG1109, patients with distant metastases received salvage chemotherapy. However, the absence of immune checkpoint inhibitors, which enhance efficacy in metastatic esophageal cancer when combined with chemotherapy (27–32), likely limited survival benefits in the chemoradiotherapy group (33, 34).

Additionally, neoadjuvant chemoradiotherapy may increase postoperative and non-cancer-related mortality, contributing to its modest survival improvements. The JCOG1109 trial reported significantly higher non-cancer-related deaths with chemoradiotherapy versus triplet chemotherapy (25.8% vs. 9.5%), consistent with prior studies (20, 35).

Elevated non-cancer mortality, particularly from lung and cardiac causes, may stem from radiation fields. The JCOG1109 trial employed elective lymph node irradiation covering most mediastinal lymph nodes for middle and lower thoracic tumors, increasing radiation exposure to adjacent organs and associated risks (36, 37). Conversely, restricted fields for upper thoracic disease likely reduced cardiac exposure, contributing to a favorable HR of 0.68 for this subgroup.

Current practice favors involved field irradiation for esophageal cancer, as it minimizes radiation to organs at risk while maintaining survival outcomes comparable to elective nodal irradiation (38–40). When involved-field irradiation was used, no significant differences emerged in mortality due to neoadjuvant therapy side effects (7.0% vs. 3.1%, P = 0.684), postoperative complications (12.3% vs. 6.2%, P = 0.355), other diseases (5.3% vs. 1.6%, P = 0.622), or unknown causes (3.5% vs. 6.2%, P = 0.684) between chemoradiotherapy and chemotherapy cohorts (41).

Critically, JCOG1109 showed no significant overall survival improvement for triplet chemotherapy over chemoradiotherapy (HR = 0.80, 95% CI: 0.59-1.10). Limitations in the chemoradiotherapy group, including elective nodal irradiation protocols, surgical techniques, and salvage therapies, further complicate its clinical application. Among these challenges, integrating immunotherapy emerges as particularly significant.

Summary: Intensive neoadjuvant chemotherapy enhances survival through superior systemic control of distant metastases and lower non-cancer mortality compared to chemoradiotherapy, despite higher locoregional recurrence rates which are treatable with salvage radiotherapy. Limitations of radiation fields and lacking of immunotherapy in salvage settings further impacted chemoradiotherapy outcomes.

## Neoadjuvant immunotherapy improves pathological complete response rates

The ESCORT-NEO/NCCES01 trial compared neoadjuvant camrelizumab plus chemotherapy versus neoadjuvant chemotherapy alone (42). Results revealed that camrelizumab with albumin-bound paclitaxel and cisplatin significantly improved pathological complete response (pCR) rates (28.0% vs. 4.7%, P < 0.0001). Although this pCR exceeded the 19.8% reported for triplet chemotherapy (fluorouracil, cisplatin, and docetaxel), direct comparison was not performed. Grade ≥3 treatment-related adverse events during neoadjuvant treatment occurred in 34.1% versus 28.8% between immunochemotherapy and chemotherapy groups, with postoperative complication rates of 34.2% and 32.0%.

The REVO trial further compared pCR rates between immunochemotherapy (camrelizumab, albumin-bound paclitaxel, and cisplatin) and chemoradiotherapy (36–40 Gy plus albumin-bound paclitaxel and cisplatin). The immunochemotherapy cohort achieved higher pCR (40.6% vs. 35.7%). Grade ≥3 treatment-related adverse events were 22% versus 31.8% between immunochemotherapy and chemoradiotherapy groups before surgery, and 28.1% vs. 21.4% after surgery. Notably, the 40.6% pCR rate of the REVO trial exceeded the 28.0% of the ESCORT-NEO/NCCES01 trial (42). This enhanced pCR correlated with superior 2-year overall survival (81.3% vs 71.3%; HR = 1.57, 95% CI: 1.26-1.96; P < 0.001) and disease-free survival (73.9% vs 63.4%; HR = 1.37, 95% CI: 1.11-1.69; P < 0.001) (43).

However, pCR rates for immunochemotherapy vary considerably (16.7%-57.1%) (44, 45), with 26.9% (95% CI, 16.7%-38.3%) treatment-related severe adverse events. This heterogeneity prompts questions about the impact of immunotherapy when combined with chemoradiotherapy. The Palace-1 trial evaluated preoperative pembrolizumab plus chemoradiotherapy (41.4 Gy plus carboplatin and paclitaxel) for resectable esophageal squamous cell carcinoma (46). This regimen proved safe, did not delay surgery, and achieved 55.6% pCR. Similarly, NEOCRTEC1901 reported higher pCR with toripalimab plus chemoradiotherapy (44 Gy plus paclitaxel and cisplatin) versus chemoradiotherapy alone (50% vs. 36%, P = 0.19) (47). Collectively, neoadjuvant immunotherapy plus chemoradiotherapy consistently achieve pCR >50%, which is higher than immunochemotherapy (48, 49). Safety profiles remain comparable, with no significant differences in grade 3/4 adverse events or postoperative complications versus neoadjuvant chemoradiotherapy.

Summary: Neoadjuvant immunotherapy significantly enhances pCR rates, whether added to chemotherapy or chemoradiotherapy, while maintaining comparable safety profiles to standard neoadjuvant approaches.

## Pathological complete response rate is not a prognostic factor

Despite the high pCR rates observed with immunotherapy combined with chemoradiotherapy, these rates did not correlate with improved survival outcomes. Although the immunochemotherapy demonstrated a significantly lower pCR rate compared to immunotherapy plus chemoradiotherapy (32.3% vs. 52.1%, P = 0.004), the 2-year overall survival rates were similar (84.42% vs. 81.70%, P = 0.860), as were the 2-year disease-free survival rates (83.21% vs. 80.47%, P = 0.839) (50).

The JCOG1109 trial reinforced these findings, reporting a substantially higher pCR rate in the neoadjuvant chemoradiotherapy group (38.5%) compared to the triplet chemotherapy (19.8%) and doublet chemotherapy (2.0%) groups (24). Nevertheless, this elevated pCR rate did not translate into improved overall survival relative to either the triplet or doublet chemotherapy cohorts. In contrast, the triplet chemotherapy group did exhibit an overall survival benefit over the doublet group.

Similarly, the NeoRes trial revealed minimal differences in long-term survival between the neoadjuvant chemoradiotherapy and chemotherapy groups, despite a significant disparity in pCR rates (28% vs. 9%) (20). The CMISG1701 study also documented a higher pCR rate in the chemoradiotherapy group (27.7% vs. 2.9%), yet no significant difference in survival outcomes was observed (HR = 0.82, P = 0.28) (21).

Collectively, these studies suggest that pCR may not be a reliable prognostic indicator for long-term outcomes when comparing different preoperative treatment strategies (51). In the absence of comprehensive long-term survival data, the pCR rates associated with various neoadjuvant therapies fail to clarify whether chemoradiotherapy combined with immunotherapy is superior to triplet chemotherapy or immunochemotherapy. This leads to a pivotal question: What is the clinical significance of achieving pCR rates exceeding 50% through the combination of chemoradiotherapy and immunotherapy?

Summary: Superior pCR rates achieved with various neoadjuvant chemoradiotherapy or immunotherapy combinations do not correlate with improved overall survival compared to regimens yielding lower pCR rates, challenging the value of pCR as a prognostic indicator for long-term outcomes.

## Organ preservation after neoadjuvant immunotherapy plus chemoradiotherapy

The clinical significance of achieving high pCR rates following neoadjuvant immunotherapy combined with chemoradiotherapy lies in organ preservation. The SANO trial, a phase III multicenter stepped-wedge cluster randomized controlled trial, compared active surveillance with standard surgical intervention for locally advanced esophageal cancer patients who achieved a clinical complete response (cCR) after neoadjuvant chemoradiotherapy (41.1 Gy plus carboplatin and paclitaxel) (52). Patients with cCR were randomized to either active surveillance (with salvage surgery upon local recurrence) or immediate standard surgical treatment. Of the 309 patients evaluated, 198 were allocated to active surveillance and 111 underwent standard surgery. With a median follow-up of 38 months, overall survival in the active surveillance cohort was non-inferior to that in the surgery group (HR = 1.14, 95% CI: 0.74-1.78; P = 0.55), indicating that esophageal preservation is a feasible alternative for patients attaining cCR after neoadjuvant chemoradiotherapy.

Quality of life was significantly better in the active surveillance group at both 6 months (P = 0.002) and 9 months (P = 0.007). Surgical outcomes were comparable, with R1 resection rates of 2% in both groups and 90-day postoperative mortality rates of 4% and 5%, respectively. Therefore, organ preservation has emerged as a shared goal for clinicians and patients, particularly as neoadjuvant chemoradiotherapy combined with immunotherapy achieves pCR rates exceeding 50%, suggesting that a substantial proportion of patients may avoid esophagectomy.

The critical challenge for esophagus preservation is accurately identifying patients with cCR. The preSANO study outlined a multimodal assessment protocol (1): Deep bite-on-bite biopsies under endoscopic ultrasonography to assess pathological regression in the primary tumor (2), Fine-needle aspiration of suspicious lymph nodes to evaluate regional lymph node status, and (3) 18-fluorodeoxyglucose positron emission tomography/computed tomography to detect potential distant metastases (53).

These methods yielded false-negative rates of 10% (95% CI: 4%–23%) for locoregional recurrence and 15% (95% CI: 7%-28%) for distant metastases. To improve accuracy in assessing primary tumor regression and lymph node status, incorporating magnetic resonance imaging alongside 18-fluorodeoxyglucose positron emission tomography/computed tomography is recommended, as observed morphological changes can aid in identifying pCR (54–56). Additionally, circulating tumor DNA analysis may enhance the performance of positron emission tomography/computed tomography, given that circulating tumor DNA-positive patients exhibit higher rates of distant metastases (15.1% vs. 3.3%) (53).

While effective, the complexity and cost of these detection methods make them impractical for all patients following chemoradiotherapy plus immunotherapy. Selective application is therefore advised for populations with a high likelihood of achieving cCR, necessitating the identification of predictive markers for treatment response.

Currently, programmed cell death ligand 1 expression is a commonly used biomarker. However, its predictive value for cCR in esophageal cancer clinical trials remains inconsistent (27–32). Furthermore, optimal cutoff values for tumor proportion score and combined positive score have yet to be firmly established, warranting caution in relying solely on programmed cell death ligand 1 to predict cCR.

Summary: High pCR rates from neoadjuvant immunotherapy plus chemoradiotherapy enable organ preservation strategies, as evidenced by the SANO trial where active surveillance was non-inferior to surgery for cCR patients and improved quality of life. However, accurately identifying candidates for preservation remains challenging due to limitations in current cCR assessment methods and the absence of validated biomarkers.

## Future perspectives

Based on the highest current level of evidence, neoadjuvant chemoradiotherapy remains the recommended standard approach (4–7). Following this treatment, patients are advised to undergo esophagectomy. For those seeking organ preservation, a comprehensive efficacy assessment should be conducted. If cCR is confirmed, organ preservation may be considered; otherwise, surgical intervention is indicated. Postoperatively, patients achieving should transition to surveillance, while those without pCR require adjuvant immunotherapy.

In patients failing to achieve pCR after esophagectomy, adjuvant immunotherapy is essential. The CheckMate 577 trial demonstrated this principle by randomizing patients without pCR after neoadjuvant chemoradiotherapy and surgery (2:1 ratio) to receive nivolumab or placebo (57). Nivolumab significantly improved median disease-free survival versus placebo (22.4 vs. 11.0 months; HR = 0.69, 96.4% CI = 0.56-0.86; P < 0.001), with consistent benefits across all prespecified subgroups.

However, neoadjuvant immunotherapy is increasingly utilized in both trials and clinical practice, demonstrating substantial efficacy. Given the inconsistent outcomes associated with various neoadjuvant regimens (chemotherapy, radiotherapy, and immunotherapy), optimal strategies require further evaluation. The ongoing SCIENCE trial, a prospective, multicenter, randomized phase III study, aims to address this by enrolling 420 patients with locally advanced thoracic esophageal squamous cell carcinoma (58). Participants will be randomized (1:1:1) into three groups (1): neoadjuvant chemotherapy plus immunotherapy (2), neoadjuvant chemoradiotherapy plus immunotherapy, or (3) neoadjuvant chemoradiotherapy alone. This trial is expected to provide high-level evidence for this clinical dilemma.

Future research should prioritize identifying robust predictive biomarkers. Emerging evidence indicates that CD8+ Tex-SPRY1 cells enhance antitumor immunity by promoting a pro-inflammatory macrophage phenotype and supporting B cell function (59). Moreover, elevated levels of CD8+ and CD4+ tumor-infiltrating lymphocytes are associated with improved therapeutic outcomes (60). Additionally, patients with a high tumor mutation burden may derive greater survival benefits from immunotherapy (61). These biomarkers hold promise for predicting treatment response and enabling risk stratification.

Summary: The future treatment involves refining the current standard (chemoradiotherapy followed by surgery) by integrating organ preservation for cCR patients and adjuvant immunotherapy for non-pCR cases. While the SCIENCE trial seeks to define the optimal neoadjuvant approach incorporating immunotherapy; crucially, identifying reliable predictive biomarkers is essential for advancing personalized therapy.

## Conclusion

Neoadjuvant triplet chemotherapy demonstrates superior systemic oncological control, reducing the incidence of distant metastases and thereby contributing to improved overall survival. In contrast, neoadjuvant chemoradiotherapy enhances locoregional control, evidenced by reduced local recurrent lesions, higher pCR rates, increased R0 resection rates, and decreased lymph node metastasis frequency, though without conferring overall survival benefits. Neoadjuvant immunotherapy combined with either chemotherapy or chemoradiotherapy achieves significantly higher pCR rates compared to chemotherapy or chemoradiotherapy alone. Critically, however, this elevated pCR rate does not correlate with improved overall survival or disease-free survival.
